# Case Report: Fournier's gangrene caused by *Gleimia hominis*

**DOI:** 10.3389/fmed.2026.1933680

**Published:** 2026-09-23

**Authors:** Jin Woong Suh, Jun Seong Son, Soo-youn Moon

**Affiliations:** Department of Internal Medicine, Kyung Hee University School of Medicine, Seoul, Republic of Korea

**Keywords:** *Actinomyces*, Fournier's gangrene, *Gleimia hominis*, MALDI-TOF MS, necrotizing soft tissue infection

## Abstract

**Background:**

Fournier's gangrene is typically polymicrobial necrotizing fasciitis of the perineal/genital regions. *Gleimia hominis* (Actinomycetaceae) is a rare anaerobic Gram-positive rod whose clinical role remains poorly defined.

**Case presentation:**

A 71-year-old man with Parkinson disease, dementia, and chronic obstructive pulmonary disease was transferred for worsening fever and perineal swelling despite 6 days of piperacillin-tazobactam. Computed tomography showed a rim-enhancing fluid collection extending from the left lower abdominal wall to the left hemiscrotum with gas formation. Fournier's gangrene was diagnosed, and empiric meropenem was started together with surgical incision and drainage. *G. hominis* was isolated from the abscess culture and identified by matrix-assisted laser desorption/ionization time-of-flight mass spectrometry. Antimicrobial therapy was de-escalated to ampicillin-sulbactam, followed by oral amoxicillin-clavulanate, with clinical improvement.

**Conclusion:**

This case highlights the pathogenic potential of *G. hominis* in necrotizing soft tissue infection and the value of precise microbiologic identification for antimicrobial de-escalation.

## Introduction

Fournier's gangrene (FG) is a rare but rapidly progressive form of necrotizing fasciitis involving the deep and superficial fascial planes of the perineal, anal, scrotal, and genital regions (1, 2). It predominantly affects males between 50 and 79 years of age (3) and is typically polymicrobial, with an average of four different microorganisms per case. The most common being *Streptococcus, Staphylococcus*, and *Escherichia* species (4). The diagnosis of FG is primarily clinical but may be supported by laboratory testing and imaging modalities such as ultrasound, computed tomography (CT), or magnetic resonance imaging (2). Once FG is diagnosed, urgent administration of broad-spectrum antibiotics and surgical debridement are the cornerstones of management (4).

*Gleimia hominis* is a Gram-positive anaerobic rod formerly classified within the genus *Actinomyces*. Its role in human disease is incompletely understood, but modern diagnostic methods such as MALDI-TOF mass spectrometry have improved recognition of unusual anaerobic pathogens. Only a few cases of necrotizing fasciitis caused by *Actinomyce* spp. have been reported, including a case of FG caused by *Gleimia europaea* (formerly *Actinomyces europaea*) (5) and a case of thigh necrotizing fasciitis caused by *A. europaea* and *Clostridium innocuum* (6).

We report a case of Fournier's gangrene caused by *G. hominis*, and review the clinical implications of this unusual microbiologic finding.

## Case description

A 71-year-old male was transferred to our hospital for further assessment of fever and perineal swelling. He resided in a nursing facility and had underlying Parkinson's disease, dementia, and chronic obstructive pulmonary disease. Despite six days of intravenous piperacillin/tazobactam (4.5 g every 8 h) at the referring hospital, his cutaneous swelling, warmth, and erythema progressively worsened, suggesting inadequate source control or involvement of less common pathogens.

Upon admission, his vital signs were as follows: blood pressure 111/69 mmHg, heart rate 78 beats/min, respiratory rate 20 breaths/min, and body temperature 36.5 °C. The skin over the lower abdomen, perineum and scrotum was erythematous, warm and tender to palpation. Laboratory findings were as follows: WBC 5,870/μL (78.4% segmented neutrophils), hemoglobin 10.0 g/dL, hematocrit 30.4%, platelet count 234,000/μL, C-reactive protein (CRP) 5.63 mg/dL, aspartate aminotransferase (AST)/alanine aminotransferase (ALT) 33/14 U/L, and blood urea nitrogen (BUN)/creatinine (Cr) 40/1.37 mg/dL. Urinalysis revealed pyuria and hematuria [red blood cells 10–29/high-power field (HPF), white blood cells >50/HPF].

Contrast-enhanced abdominopelvic computed tomography (CT) performed in the emergency department demonstrated a rim-enhancing fluid collection measuring approximately 15 cm × 6 cm (coronal view), extending from the left lower anterior abdominal wall to the left inguinal region and left hemiscrotum, with linear gas formations and multiple bladder stones (Figure 1). Fournier's gangrene was diagnosed. Although the patient seemed stable, his infection was getting worse despite broad-spectrum antibiotic therapy for 6 days in the previous hospital, and high prevalence of ESBL-producing pathogens, meropenem was initiated as empirical therapy. Surgical incision and drainage were performed, and intraoperative abscess cultures were obtained.

**Figure 1:**
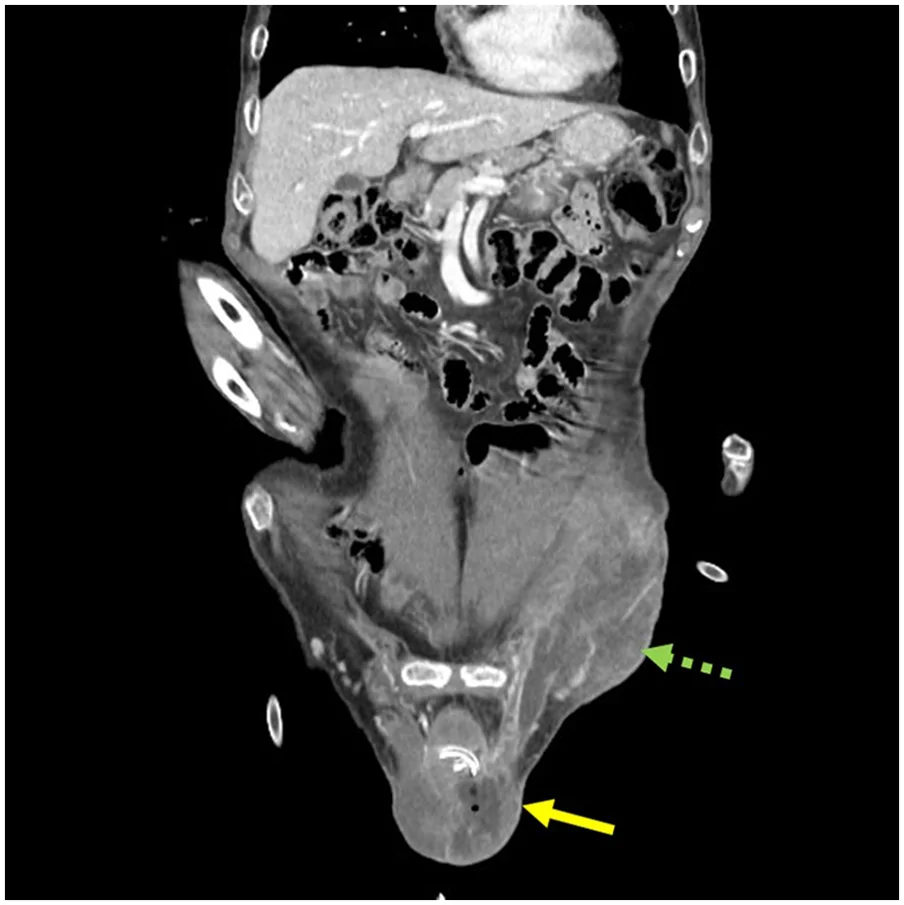
Contrast-enhanced abdominopelvic CT showing a rim-enhancing fluid collection measuring approximately 15 cm × 6 cm (green dotted arrow) with gas formation (yellow arrow), extending from the left lower anterior abdominal wall to the left hemiscrotum.

No purulent material was observed at the scrotal and abdominal wall incisions; instead, clear serous fluid was drained. An epidermal inclusion cyst (1 cm × 1 cm) was excised from the abdominal wall.

Empirical meropenem (1 g every 8 h) was continued, and a second incision and drainage procedure with concurrent bladder stone removal was performed 1 week later.

Blood cultures obtained on admission remained negative, whereas the abscess culture yielded *G. hominis* on hospital day 8, as identified by matrix-assisted laser desorption-ionization time-of-flight mass spectrometry (MALDI-TOF MS) (Figure 2). Based on the culture results and susceptibility testing, antimicrobial therapy was changed to ampicillin/sulbactam (3 g every 8 h).

**Figure 2:**
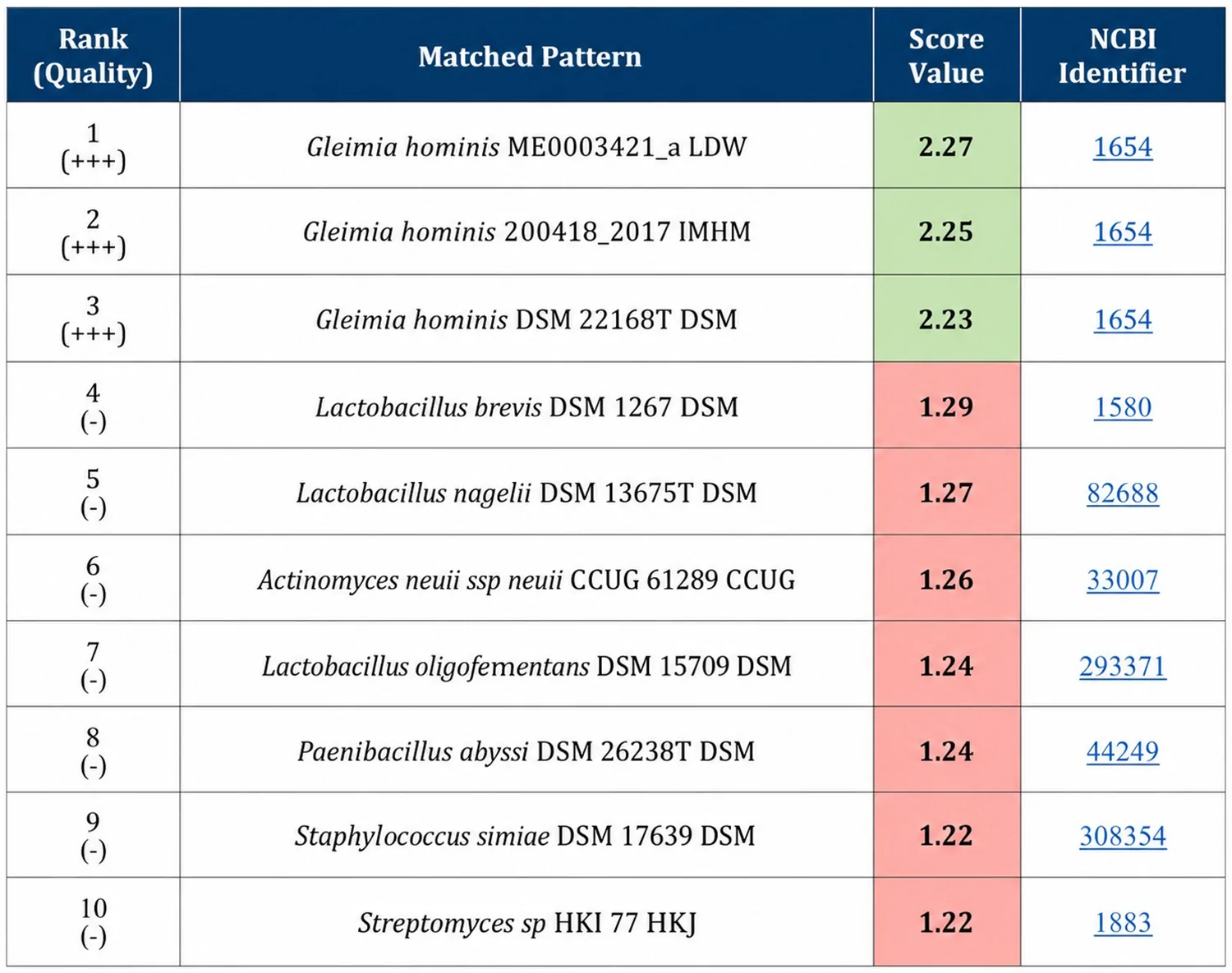
Identification of *Gleimia hominis* by MALDI-TOF MS.

The perineal wound healed well, and the patient's general condition stabilized. The patient was transferred back to his nursing facility, and oral amoxicillin/clavulanate was continued there.

The clinical course of the patient is summarized in Figure 3.

**Figure 3:**
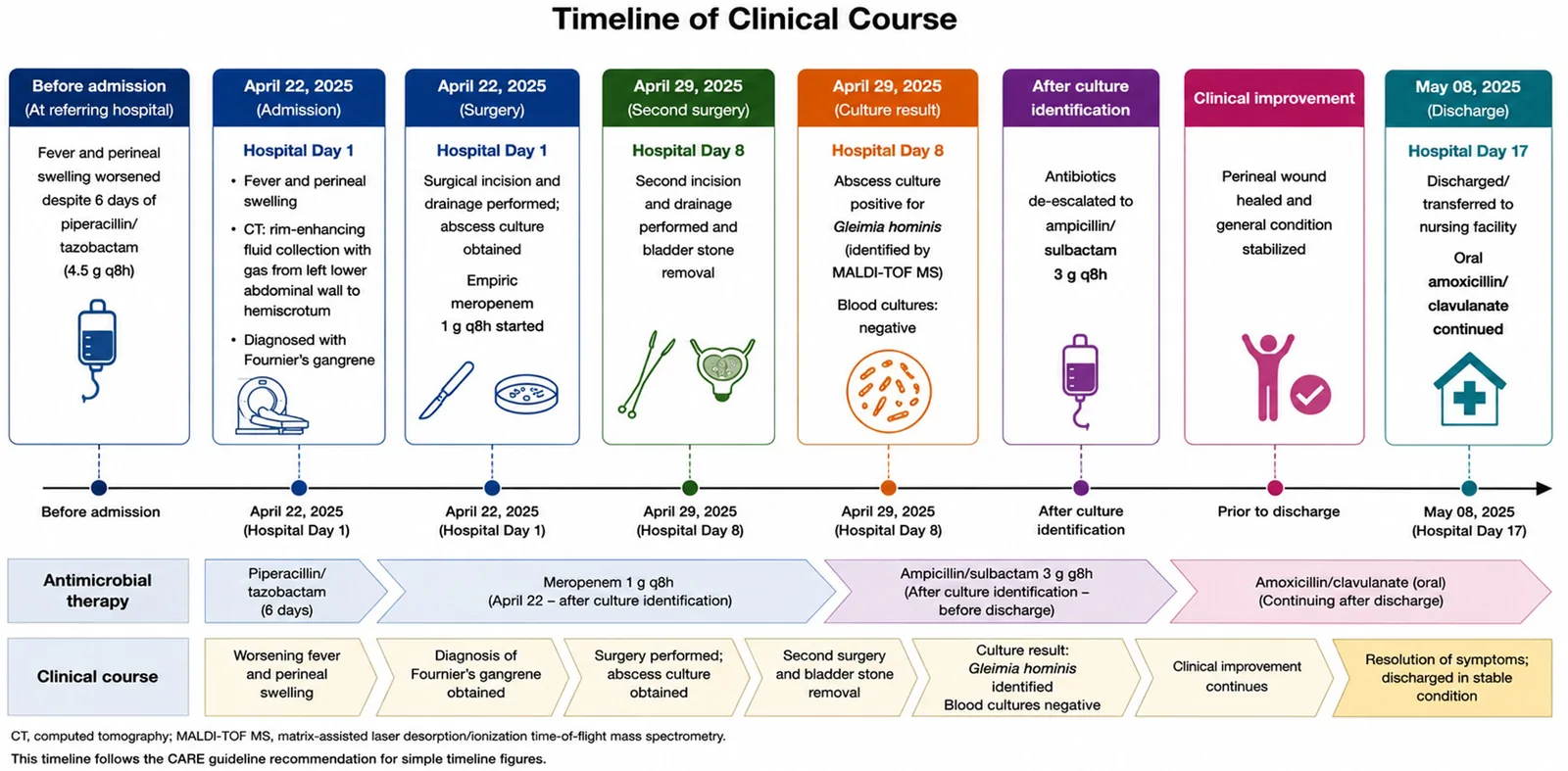
Timeline of the patient's clinical course during hospitalization.

## Discussion

Fournier's gangrene (FG) is a rare but rapidly progressive form of necrotizing fasciitis that affects the deep and superficial fascial planes of the perineal, anal, scrotal, and genital regions (1, 2). It is often a polymicrobial infection (58.4%) that spreads along the fascial planes due to thrombosis of subcutaneous vessels, tissue ischemia, superinfection, and necrosis (1). This infection demonstrates a strong male predominance, and its incidence has increased with population aging and the rising prevalence of diabetes (2). It is often a polymicrobial infection with an average of four microorganisms per case, including *Streptococcus, Staphylococcus*, and *Escherichia* species (4). The diagnosis of FG is primarily clinical but may be supported by laboratory testing and imaging modalities such as ultrasound, CT, or magnetic resonance imaging (2).

*Gleimia hominis* is a Gram-positive anaerobic rod formerly classified within the genus *Actinomyces* (7). Members of *Actinomyces* are part of the normal flora at various body sites, including the oral cavity, pharynx, and genitourinary tract (7). Advances in microbiological diagnostics, such as MALDI-TOF MS and 16S rRNA gene sequencing, have enabled more precise identification and differentiation of *Actinomyces*-like organisms (7). To date, approximately 26 Actinomyces species have been reported to cause human disease (8). In this case, the use of MALDI-TOF MS enabled precise identification of this uncommon organism, which may be otherwise misidentified or overlooked using conventional methods. The most common species associated with human disease include *Actinomyces israelii*, *Actinomyces europaeus* (renamed *Gleimia europaea*), *Actinomyces meyeri* (renamed *Schaalia meyeri*), *Actinomyces odontolyticus* (renamed *Schaalia odontolytica*), *Actinomyces viscosus*, and *Actinomyces naeslundii* (6).

The most common clinical presentations of Actinomyces infections are sebaceous cysts and pilonidal abscesses (9). Genitourinary tract actinomycosis is the second most common clinical form of actinomycosis after cervicofacial involvement (10). *A. israelii* is one of the most common species involved in pelvic actinomycosis and is often associated with intrauterine devices (IUDs) (10).

A similar case involving *G. europaea* (formerly *Actinomyces europaea*) has been reported, supporting the pathogenic potential of this genus in necrotizing infections. A previous report described FG caused by *G. europaea* as the sole pathogen; the patient was a 61-year-old female with type 2 diabetes mellitus and hypertension, in whom *G. europaea* was isolated only from pus cultures, whereas blood cultures were negative (5). The patient was treated with cefoperazone-sulbactam empirically then changed to piperacillin-tazobactam. She survived after antibiotic therapy with multiple staged debridements and graft skin closure surgery (5). In the present case, the patient was an elderly man residing in a nursing facility with multiple chronic comorbid conditions. Similar to a previously reported *G. europaea* case, *G. hominis* was the sole pathogen isolated from the abscess, and blood cultures showed no growth.

There is also a report of *G. hominis* isolated from a wound culture of an 89-year-old female, with no further clinical information provided (11). Taken together, these findings support the pathogenic potential of *Gleimia* species, including *G. hominis*, in severe soft tissue and necrotizing infections, rather than merely representing colonizers or contaminants.

In general, Actinomyces are resistant to metronidazole, fosfomycin, trimethoprim/sulfamethoxazole, and most quinolones, but are almost uniformly susceptible to most beta-lactam antibiotics (>99% susceptibility to penicillin, amoxicillin, piperacillin-tazobactam, ceftriaxone, and carbapenems) (8). Therefore, accurate identification is critical to guide appropriate antimicrobial de-escalation. In this case, the patient was treated with meropenem as empirical therapy, considering a polymicrobial infection with potential anaerobes. After identification of *G. hominis*, antimicrobial therapy was de-escalated to intravenous ampicillin/sulbactam and oral amoxicillin/clavulanate, which was consistent with the known susceptibility profile of Actinomyces-related organisms.

To the best of our knowledge, this is the first report of FG associated with *G. hominis*. This case adds to the limited clinical data on *G. hominis* and supports its role as a true pathogen in necrotizing soft tissue infections. We were unable to perform 16S rRNA sequencing for further identification, which is a limitation of this report. Clinicians should be aware that *G. hominis* and other unusual anaerobic organisms may be involed in FG and similar necrotizing infections and should ensure that empirical regimens provide adequate anaerobic coverage while allowing subsequent de-escalation based on microbiological results.

## Data Availability

The original contributions presented in the study are included in the article/Supplementary Material, further inquiries can be directed to the corresponding author/s.
